# Prevalence of Eosinophilic Esophagitis and Lymphocytic Esophagitis in Adults with Esophageal Food Bolus Impaction

**DOI:** 10.1155/2016/9303858

**Published:** 2016-07-28

**Authors:** Kotryna Truskaite, Aldona Dlugosz

**Affiliations:** Department of Medicine, Huddinge, and Center for Digestive Diseases, Karolinska Institutet and Karolinska University Hospital, 14186 Stockholm, Sweden

## Abstract

*Background*. The relation of esophageal food bolus impaction (FBI) to eosinophilic esophagitis (EoE) and lymphocytic esophagitis (LyE) is unclear. The aim of this study was to determine the prevalence of EoE and LyE among adults with FBI.* Methods*. In this retrospective study we analyzed data from all patients referred for gastroscopy during the past 5 years, because of a present or recent episode of FBI.* Results*. We found 238 patients with FBI (median age 51 (17–96), 71% males). Endoscopic therapy was required in 143 patients. Esophageal biopsies were obtained in 185 (78%) patients. All biopsies were assessed for numbers of eosinophils and lymphocytes. EoE was found in 18% of patients who underwent biopsy. We found 41 patients (22%) who fulfilled the criteria for both EoE and LyE (EoE/LyE). LyE was found in the 9% of patients with FBI. EoE together with EoE/LyE was the leading cause of FBI in patients ≤50 years (64%). GERD was the leading cause of FBI among patients older than 50 years (42%).* Conclusions*. Our study showed that EoE was the leading cause of FBI in particular among young adults. Our study highlights the need for esophageal biopsies in any patient with FBI.

## 1. Introduction

Esophageal food bolus impaction (FBI) is relatively common in clinical practice with an estimated annual incidence of 13 episodes per 100,000 [1]. Previous studies indicated that the most common aetiologies of FBI were anatomical abnormalities such as Schatzki rings and peptic strictures [2]. Other less frequent causes were dysmotility disorders, malignancy, and amyloidosis [3, 4].

During the last decade an emerging number of published papers show a strong association of FBI with eosinophilic esophagitis (EoE) [5–8]. EoE is defined as a chronic, immune/antigen-mediated, esophageal disease characterized clinically by symptoms related to esophageal dysfunction and histologically by an eosinophil-predominant inflammation (≥15 eosinophils per high power field (hpf)) in the esophageal mucosa [9]. Endoscopic abnormalities in patients with EoE include esophageal rings, strictures, narrow-caliber esophagus, linear furrows, white plaques or exudates, and edema [10]. However, there are no pathognomonic signs for EoE since these endoscopic findings have also been described in other esophageal disorders. Moreover, endoscopic appearances may be normal in 10–25% of EoE patients [11].

A few studies, although based on small numbers of patients, show that 33% to 54% of the patients presenting with esophageal FBI have EoE as an underlying cause [5, 7]. The prevalence of FBI episodes, occurring as a first symptom in EoE patients, ranges from 25% to 100% [3, 5]. The prevalence of FBI has increased over the last 15 years correlating with an increased prevalence of EoE and a reduction in age of presentation and peptic related strictures [6].

Lymphocytic esophagitis (LyE) was first described as an independent entity in 2006 by Rubio et al. [12]. LyE patients clinically present with dysphagia, abdominal pain, heartburn, and nausea [13]. The diagnosis of LyE is considered when more than 40 intraepithelial lymphocytes/hpf are present and there are no or only occasional CD15+ intraepithelial granulocytes [12, 14]. Endoscopic features of LyE can be similar to EoE including esophageal rings, furrows, exudates, narrow lumen, or stenosis but in one-third of patients the esophageal mucosa appears macroscopically normal [13]. To this date there are no studies published on the prevalence of LyE in patients with FBI.

The aim of our study was to identify clinicopathological features of adults presenting with FBI at a tertiary care center. The secondary aim was to determine the prevalence of EoE and LyE among adults with FBI.

## 2. Material and Methods

**Figure 1:**
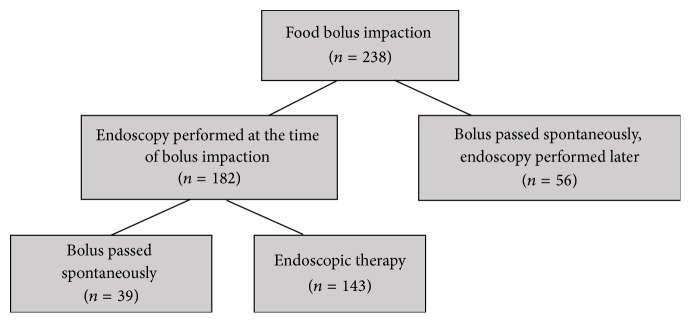
Flowchart showing the endoscopy outcome.

We analyzed adult patients referred to the Endoscopy Unit at Karolinska University Hospital, Stockholm, Sweden, because of present or recent symptoms suggesting food bolus impaction (FBI). Patients with impacted items other than food as well as patients with dysphagia without episodes of FBI were excluded. In the period from August 2011 to April 2016 we identified 238 such patients by searching the electronic medical record system Take Care by ICD codes T18.1 and R13. Gastroscopy was performed at the time of food bolus impaction in 182 patients (76%). In 39 out of 182 patients, food bolus had passed spontaneously by the time of gastroscopy. In 143 patients (60%) endoscopic therapy was required by either pushing the food bolus into the stomach using push technique or removing it using a retrieval net. In 56 patients the food bolus had passed spontaneously before medical contact and these patients were all referred for gastroscopy later by their general practitioner (Figure 1).

One hundred thirty-four patients were followed up in the outpatients' department. Gastroscopies with biopsies from the distal, middle, and proximal esophagus as well as from the antrum and duodenum to exclude other pathologies of the gastrointestinal (GI) tract were performed by experienced endoscopists. In line with the established guidelines [9, 11], all patients with a histopathological finding of more than 15 eosinophils/hpf were treated with proton pump inhibitors (PPI) 40 mg daily and had a follow-up endoscopy after 6–12 weeks of treatment.

Experienced pathologists analyzed all biopsies according to standard routines and all biopsies were assessed for numbers of eosinophils using chromatic staining and lymphocytes using immunohistochemistry for the pan-T cell marker CD3 according to standard protocols [14].

### 2.1. Study Definitions

The presence of ≥15 eosinophils/hpf in the esophageal mucosa was considered as eosinophilic esophagitis (EoE) and that with 8–15 eosinophils/hpf as eosinophilic infiltration (Eo-inf). The finding of ≥40 intraepithelial lymphocytes (IELs) per hpf was considered as lymphocytic esophagitis (LyE) and that with 20–40 IELs/hpf as lymphocytic infiltration (L-inf) [14].

Patients fulfilling criteria for both EoE and LyE (>15 eosinophils/hpf and >40 IELs/hpf) were considered as having compound eosinophilic and lymphocytic esophagitis (EoE/LyE).

The presence of ≥15 eosinophils/hpf that normalized after 6–12 weeks of PPI treatment combined with a normal pH monitoring was considered as PPI-responsive esophageal eosinophilia (PPI-REE).

### 2.2. Statistical Analysis

Descriptive statistics were used to summarize findings. Statistical analysis was done using chi-squared analysis for gender and aetiology and Mann-Whitney *U* test for age. *p* < 0.05 was considered as statistically significant.

## 3. Results

During the 5-year study period, a total of 238 patients (median age 51, range 17–96) underwent gastroscopy at Karolinska University Hospital because of a present or recent episode of food bolus impaction. The majority of patients were male (170/238 (71%) indicating a significant (*p* < 0.0001) gender difference). The most common type of impacted food was meat, occurring in 217 patients (91%). Other items included fish, bread, potatoes, carrots, oranges, and nuts.

**Figure 2:**
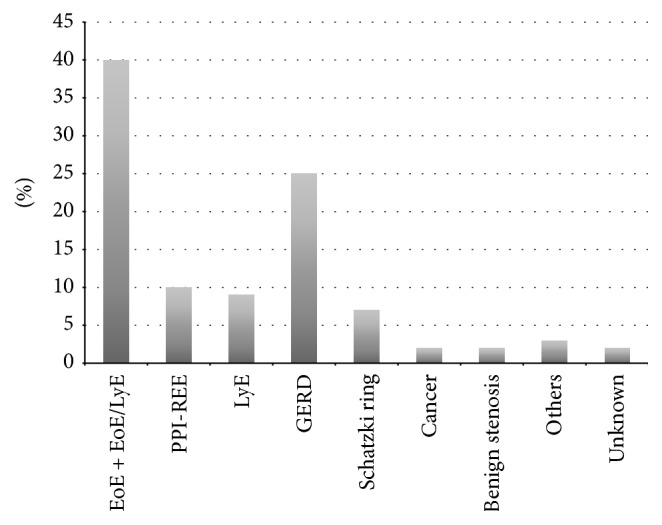
Aetiology of esophageal food impaction in 185 patients, in whom biopsies from the esophagus were obtained.

Disorders contributing to the episode of FBI included gastroesophageal reflux disease (GERD) (27%), EoE/LyE (17%), EoE (14%), PPI-REE (8%), LyE (7%), Schatzki ring (7%), esophageal stenosis (4%), cancer (1%), and other reasons (8%). Among others we grouped together cases of esophageal dyskinesia, achalasia, and esophagus diverticulum. Seventeen patients (7%) were lost to follow-up and a final diagnosis could not be established. Esophageal manometry and 24-hour pH monitoring were performed in 85 (36%) out of 238 patients. Biopsies from the esophagus were obtained in 185 patients (78%). The distribution of aetiologies confirmed by histopathology showed a prevalence of EoE of 18%, which together with compound EoE/LyE reached 40% but together with PPI-REE even 50% of causes of FBI (Figure 2). EoE together with compound EoE/LyE was the leading cause of FBI in patients ≤50 years of age (64% of patients ≤50 yrs. versus 11% of patients >50 yrs., *p* < 0.0001). GERD was the leading cause of FBI among patients older than 50 years (42% versus 12%, *p* = 0.032). For detailed characteristics see Table 1.

Information about the presence or absence of classical endoscopic features of EoE like rings, furrows, edema, or exudates was reported in 140/185 patients with obtained biopsies and in 101 patients in whom the endoscopist suspected the presence of EoE based on macroscopic findings. Rings, furrows, and/or exudates were reported in all 34 patients with EoE, 36 (88%) patients with compound EoE/LyE, 7 (44%) patients with LyE, and 11 (61%) patients with PPI-REE.

Furrows and rings in the distal part of the esophagus were reported also in 9 patients with GERD and 3 patients with Schatzki ring but particularly in patients with infiltration of eosinophils or lymphocytes (7 out of 9 patients, 2 out of 3 patients, resp.). In the remaining 53 patients, in whom biopsies were not obtained, gastroscopy showed the presence of erosive esophagitis in 17 patients, Schatzki ring in 3 patients, benign stenosis in 6 patients, and esophagus diverticula in 3 patients. In 24 patients the esophageal mucosa appeared normal during gastroscopy.

Patients with EoE (median age 34.5, range 18–73) and compound EoE/LyE (median age 35, range 17–82) were significantly younger compared to patients with other causes of FBI (*p* < 0.0001). Patients with PPI-REE (median age 47, range 32–69) were significantly younger compared to patients with GERD (median age 67.5, range 21–94, *p* = 0.0027) (Table 2).

Patients with increased number of eosinophils in the esophagus (median age 37, range 17–82) were significantly younger compared to patients without such infiltration (*p* < 0.0001) (Table 3).

Biopsies from 18/47 patients with GERD revealed inflammatory infiltrates: eosinophilic infiltration (mean 10 eosinophils/hpf, range 8–14) was found in 11 patients, lymphocytic infiltration (mean 30 IELs/hpf, range 22–39) in 6 patients, and in one patient infiltration with both eosinophils and lymphocytes was found. The subgroup of patients with GERD and eosinophilic infiltration was significantly younger than other GERD patients (median age 54.5, range 23–77, *p* = 0.016).

We found 27 cases with a lower esophageal ring (Schatzki) among our patients (11%) and in 24 cases biopsies from the esophagus were available. In 11/24 patients biopsies did not reveal any inflammatory cells but in 13 cases the Schatzki ring coexisted with inflammatory infiltration classified as EoE (4/18), EoE/LyE (2/18), PPI-REE (5/18), and L-inf (2/18).

## 4. Discussion

To the best of our knowledge, this is the first study evaluating the prevalence of compound EoE/LyE, PPI-REE, and LyE in patients presenting with food bolus impaction.

Coexistence of EoE and LyE, called overlapping phenotype, has been described before by Akiyama et al. [14]. In our study the number of compound cases exceeds the number of isolated EoE. We cannot tell if EoE/LyE presents the specific variant of EoE or if it is a separate disorder. Patients with EoE and EoE/LyE are similar in age, gender, endoscopic features, and symptoms. Further studies are required to find out if there are differences in the natural history of the two groups. Since patients with EoE and EoE/LyE were similar, we chose to group them together in order to calculate the prevalence of EoE as a cause of FBI and the combined prevalence of the two groups was 40%. These results were in line with those obtained by Desai et al. [5] and Heerasing et al. [7], although in our study we analyzed a larger number of patients.

Proton pump inhibitor-responsive esophageal eosinophilia caused 10% of episodes of FBI in our study. This is a novel observation because PPI-REE as a cause of FBI has never been mentioned in former studies, probably due to the lack of biopsies before and after PPI trial. PPI-REE refers to patients with clinical and histological features of EoE that remit with PPI treatment. Recent evidence showed that patients with PPI-REE and patients with EoE at baseline were clinically, endoscopically, and histologically indistinguishable and had a significant overlap in terms of Th2 immune-mediated inflammation and gene expression [15, 16]. In our study PPI-REE patients were significantly older compared to both EoE (*p* = 0.0016) and EoE/LyE (*p* = 0.0057) patients and only 61% presented with typical endoscopic findings like rings or furrows. We chose to present them as a separate group but with taking into account the possibility that they may represent a continuum of the same immunological mechanisms that underlie EoE but in a later phase of the disease. We increased the prevalence of EoE in our study to 50% after adding PPI-REE cases.

The other novelty of our study is the role of LyE as a cause of FBI. In 16 patients (9%) we found that the number of lymphocytes exceeded 40/hpf. Haque and Genta [17] in their retrospective population study reported that LyE affected predominantly older woman with a median age of 63 years. The median age of our patients with LyE was exactly the same but women constituted only 37.5% of studied patients with LyE. This result is probably due to the significantly higher prevalence of FBI among men in our study (*p* < 0.0001). Only 7 (44%) patients with LyE presented with rings or furrows during endoscopy. Cohen et al. [13] reported normal looking mucosa in 29.6% of patients with LyE. We found lymphocytic infiltration in 2 patients with Schatzki ring and in 5 patients with GERD. Further studies are required to explain the role of lymphocytes in these disorders. Schatzki ring is a thin, circumferential, submucosal ring that protrudes into the lumen of the distal esophagus [1]. Few former studies showed the association between the Schatzki ring and the presence of EoE [18–20]. Ohers [21] did not find such an association. In our study, among 24 patients with Schatzki ring with obtained biopsies, we found 6 cases of EoE (25%) and 5 cases of PPI-REE (21%) which indicates a possible association. Although the etiology and pathogenesis of Schatzki ring remain unclear it is frequently associated with other esophageal disorders [19] such as reflux esophagitis. Inflammatory factors can possibly lead to circular constriction of the esophageal junction [20].

Our study showed a substantial number of patients with infiltration of inflammatory cells in the esophagus (126/185, 68%). Patients with any kind of eosinophilic infiltration were significantly younger compared to those without eosinophils. It has been known that GERD can be associated with mild eosinophilic infiltration, usually less than 7 eosinophils/hpf, of the squamous epithelium in the distal esophagus [22]. In our study GERD patients with eosinophilic infiltration were significantly younger than patients without such infiltration. At the moment we cannot explain these phenomena. We cannot exclude that disorders causing esophageal FBI have a common inflammatory, still unknown denominator.

Our study has several limitations. Being a retrospective study has its limitations like potential selection bias as well as the effect of missing data. The present study was performed in a tertiary care center and patients undergoing endoscopy may not represent the community population as a whole.

Another limitation is the fact that we obtained biopsies only in a subgroup of patients. It is very important to find the cause of bolus impaction in order to start appropriate treatment and to prevent recurrence of impaction. The European Society of Gastrointestinal Endoscopy strongly recommends diagnostic workup, including histological evaluation, for potential underlying disease in cases of FBI [2]. There are very few studies on the histologic findings in the case of bolus impaction [3, 7, 8, 23]. In those studies, the number of gastroscopies with biopsies varied from 29 to 98. Our 185 cases present a substantial number of patients.

By consensus, an eosinophilic infiltrate of ≥15 eosinophils/hpf suggests the diagnosis of EoE [9]; however the distribution of esophageal eosinophilia is often patchy [24, 25]. Patients with EoE often demonstrate eosinophilia in both distal and proximal biopsies, although proximal biopsies vary in eosinophil density on a patient-to-patient basis. Endoscopists should focus their biopsies towards areas of exudates and furrows as these areas represent increased eosinophil infiltrate [26]. Nielsen et al. [27] demonstrated that four to six biopsy fragments from the distal and proximal esophagus should be submitted to optimize the chances of achieving the morphologic criteria for a diagnosis of EoE and that the field is not increased beyond six biopsy fragments. To this date we have no established guidelines regarding biopsy protocol in LyE; however, the fact that the endoscopic appearances may be normal in up to 25% of EoE patients and 30% of LyE patients supports the recommendation for biopsy acquisition in all patients with food bolus impaction.

In conclusion, our study showed that EoE was the leading cause of food impaction in particular among young adults. We would like to emphasize that esophageal biopsies from the distal and proximal esophagus with assessment of both eosinophils and lymphocytes should be obtained during endoscopy for any patient with food bolus impaction.

## Figures and Tables

**Table 1:** Aetiology of esophageal food impactions in all 238 patients and in 185 patients with obtained biopsy samples from the esophagus.

	All patients *n* = 238	Patients in whom biopsies were obtained *n* = 185	Patients in whom biopsies were obtained *n* = 185		
			≤50 years		>50 years
	*n* (%)	*n* (%)	*n* = 102		*n* = 83
GERD	64 (27)	47 (25)	12	**p** ** = 0.0032**	35
EoE	34 (14)	34 (18)	29* *	**p** ** = 0.0038**	5
EoE/LyE	41 (17)	41 (22)	37	**p** ** < 0.0001**	4
LyE	16 (7)	16 (9)	7		9
PPI-REE	18 (8)	18 (10)	12		6
Isolated Schatzki ring^a^	16 (7)	13 (7)	1		12
Cancer	3 (1)	3 (2)	0		3
Esophagus stenosis^b^	9 (4)	3 (2)	1		2
Others^c^	20 (8)^c^	6 (3)	2		4
No underlying diagnosis	17 (7)	4 (2)	1		3

^a^Schatzki ring without signs of EoE, LyE, or erosive esophagitis.

^b^Posttraumatic or postsurgical.

^c^Esophagus diverticula *n* = 4, dyskinesia due to other diseases *n* = 7, presbyesophagus *n* = 7, and achalasia *n* = 2.

GERD: gastroesophageal reflux disease, EoE: eosinophilic esophagitis, LyE: lymphocytic esophagitis, EoE/LyE: compound eosinophilic esophagitis and lymphocytic esophagitis (patients fulfilled criteria for both EoE and LyE), and PPI-REE: proton pump inhibitor-responsive esophageal eosinophilia.

**Table 2:** Characteristics of esophageal food impaction by age and gender.

Aetiology	*n*	Median age (range)	Women (%)		Men (%)
	238	51 (17–96)	68 (29)	**p** ** < 0.0001**	170 (71)
EoE	34	34.5 (18–73)	7 (3)		27 (11)
EoE/LyE	41	35 (17–82)	8 (3)		33 (14)
LyE	16	63 (40–89)	6 (3)		10 (4)
PPI-REE	18	47 (32–69)	3 (1)		15 (6)
GERD	64	67.5 (21–94)	20 (8)		44 (18)
Schatzki ring	16	66.5 (37–81)	6 (3)		10 (4)
Cancer	3	81 (69–90)	1 (1)		2 (1)
Esophagus stenosis	9	60 (29–71)	3 (1)		6 (3)
Others	20	72 (22–96)	8 (3)		12 (5)
No underlying diagnosis	17	46 (18–70)	6 (3)		11 (5)

GERD: gastroesophageal reflux disease, EoE: eosinophilic esophagitis, LyE: lymphocytic esophagitis, EoE/LyE: compound eosinophilic esophagitis and lymphocytic esophagitis (patients fulfilled criteria for both EoE and LyE), and PPI-REE: proton pump inhibitor-responsive esophageal eosinophilia.

**Table 3:** Characteristics of 185 patients with esophageal food impaction with obtained biopsy samples from the esophagus.

	*n* (%)	Median age (range)	Women (%)	Men (%)
Patients in whom biopsies from the esophagus were obtained	185 (100)	45 (17–96)	48 (26)	137 (74)

Patients with infiltration of inflammatory cells in the esophagus	126 (68)	40 (18–89)	29 (16)	97 (52)

Patients with an increased number of eosinophils in the esophagus	103 (56)	37 (17–82)	21 (11)	82 (44)
EoE	34 (18)	34.5 (18–73)	7 (4)	27 (15)
EoE/LyE	41 (22)	35 (17–82)	8 (4)	33 (18)
PPI-REE	18 (10)	47 (32–69)	3 (2)	15 (8)
GERD with Eo-inf	10 (5)	54.5 (23–77)	3 (2)	7 (4)

Patients with an increased number of lymphocytes in the esophagus	23 (12)	60 (27–89)	8 (5)	15 (8)
LyE	16 (9)	63 (40–89)	6 (3)	10 (5)
Schatzki ring with L-inf	2 (1)	64 (60–68)	1 (<1)	1 (<1)
GERD with L-inf	5 (2)	60 (27–79)	1 (1)	4 (2)

EoE ≥ 15 eosinophils/hpf.

Eo-inf 8–15 eosinophils/hpf.

LyE ≥ 40 EILs/hpf.

L-inf 20–40 IELs/hpf.

EoE/LyE > 15 eosinophils/hpf and >40 IELs/hpf.

## Abbreviations

Eo-inf: Eosinophilic infiltration
EoE: Eosinophilic esophagitis
EoE/LyE: Compound eosinophilic and lymphocytic esophagitis
FBI: Food bolus impaction
GERD: Gastroesophageal reflux disease
HPF: High power field
IELs: Intraepithelial lymphocytes
L-inf: Lymphocytic infiltration
LyE: Lymphocytic esophagitis
PPI: Proton pump inhibitors
PPI-RRE: Proton pump inhibitor-responsive esophagus eosinophilia.
